# Reconstruction of non-error magnetic hologram data by magnetic assist recording

**DOI:** 10.1038/s41598-017-12442-z

**Published:** 2017-10-09

**Authors:** Zen Shirakashi, Taichi Goto, Hiroyuki Takagi, Yuichi Nakamura, Pang Boey Lim, Hironaga Uchida, Mitsuteru Inoue

**Affiliations:** 1 Toyohashi University of Technology, 1-1 Hibarigaoka, Tempaku, Toyohashi, Aichi 441-8580 Japan; 2 JST, PRESTO, 4-1-8 Honcho, Kawaguchi, Saitama, 332-0012 Japan

## Abstract

Hologram memory is expected to be the next-generation of optical data storage technology. Bismuth-substituted yttrium iron garnet is typically used for rewritable magnetic hologram media. The diffraction efficiency of magnetic holography depends on the Faraday rotation angle, but the experimental diffraction efficiency is not as high as that expected from calculations. This difference could be caused by incomplete magnetization reversal at the recorded region. In this study, we investigated the effects of magnetic assist (MA) recording through numerical simulation and experiment to improve the diffraction efficiency and the resulting reconstructed images. The improvement of diffraction efficiency was more effective in garnet films thinner than the width of a fringe, and a suitable value of the assist magnetic field was identified for the improvement. In addition, MA recording improved the intensity of reconstructed images and broadened the non-error recording conditions to the low energy region. This technique shows promise in improving the reconstructed quality of magnetic hologram data.

## Introduction

Hologram memory is a promising data storage technology with high density, large capacity, and a fast data transfer rate^1–6^. Photopolymers have been widely used as hologram media because of their high diffraction efficiency and transparency; however, photopolymer-based hologram media are a write-once media that require light shielding. On the other hand, magnetic hologram media using magnetic garnets have shown rewritability and long-term stability. In the recording process, volumetric holograms formed by using thermomagnetic recording are recorded as the direction of magnetization in magnetic films, while reconstruction involves the use of the magneto-optical (MO) effect^7,8^. Magnetic garnet films such as Bi-substituted rare-earth iron garnet (Bi:RIG) can be used for rewritable magnetic hologram media, and we have demonstrated that a volumetric hologram can be written in these garnet films using collinear holographic recording^9,10^. However, the diffraction efficiency of these garnet films was not sufficiently high (~0.01%) for applications in actual storage devices. In previous studies, we have analysed the magnetic fringe shape of the volumetric magnetic hologram in thermomagnetic recording using the finite-element method, improved the recording conditions, and investigated the use of an artificial magnetic lattice to improve diffraction efficiency^9–14^.

**Figure 1:**
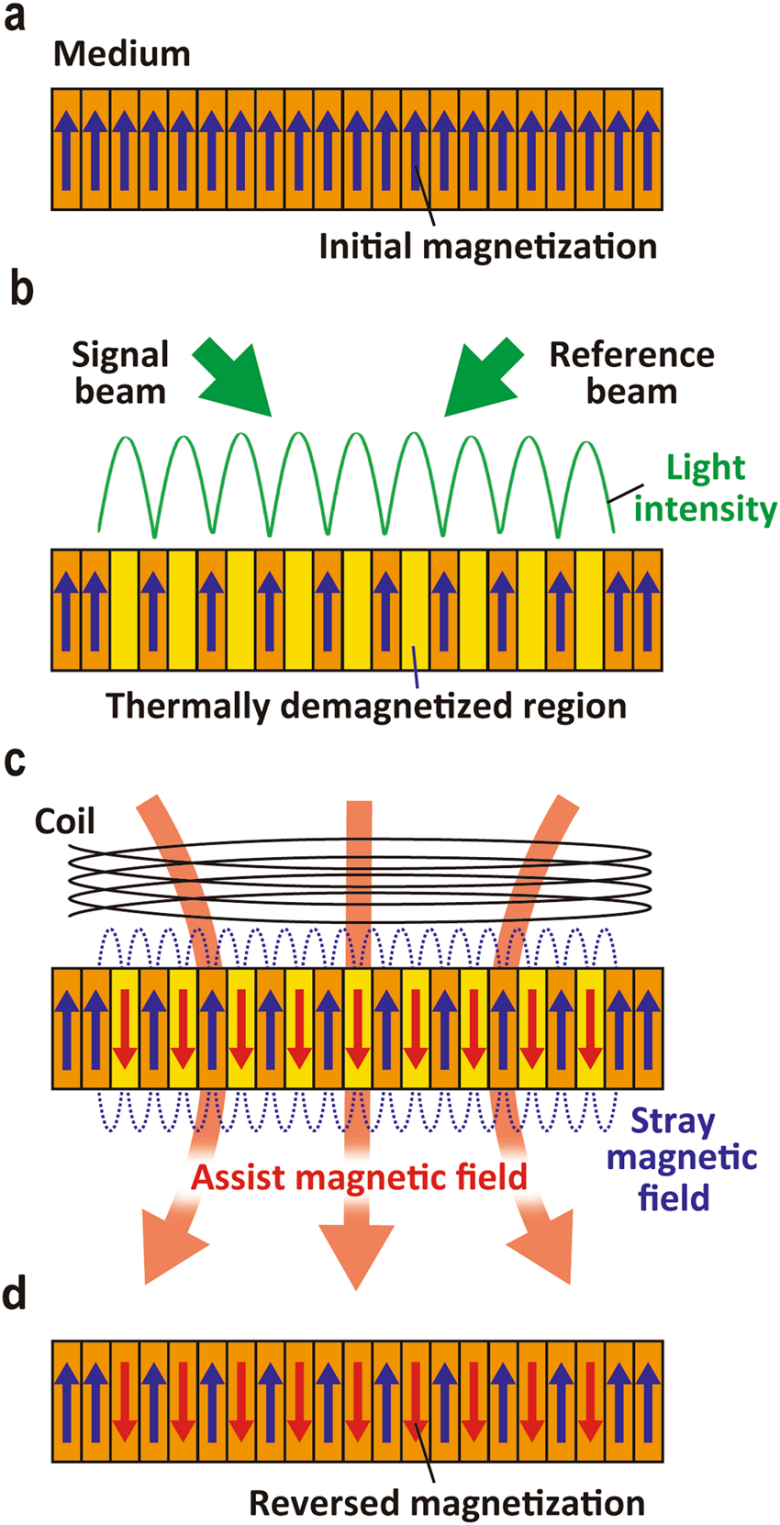
A schematic diagram of thermomagnetic recording with the use of MA. (**a**) Medium such as Bi:RIG magnetized perpendicularly in a single direction. (**b**) Two incident beams, named signal beam and reference beam, are radiated onto the medium, and the temperature distribution of interference is formed by optical absorption and heating^13^. The magnetization in the region where the temperature of the medium *T* exceeds the Curie Temperature *T*_c_ is lost and (**c**) reversed by the stray magnetic field (**d**) during the cooling process to form the magnetic hologram fringe. In the MA recording, the assist magnetic field is applied to the direction of reversed magnetization.

The diffraction efficiency could be improved by increasing the Faraday rotation, *F*, and the resulting diffraction efficiency was proportional to the square of magnetization of the magnetic film^10,15^. In magnetic holography, interference patterns were recorded as the direction of magnetization by thermomagnetic recording, as shown in Fig. 1. This recording technique uses absorbed light to form a temperature distribution in the medium that corresponds to the intensity distribution of the interference light from the signal and reference beams. The temperature rises as the amount of light energy absorbed in the medium increases. This temperature distribution yields the reversal of magnetization by stray magnetic fields in the thermally demagnetized region in which the temperature exceeded the Curie temperature, and the resulting magnetization distribution corresponds to the interference pattern^13^. However, the experimentally obtained diffraction efficiencies were lower than those expected from simulation^13^. The results suggested that the magnetization at the heated region in Bi:RIG film was not completely reversed due to a low intensity stray magnetic field.

The MA recording method is expected to be effective in improving the diffraction efficiency because the reversed magnetization intensity should be enhanced. A schematic diagram of thermomagnetic recording using MA is shown in Fig. 1. In thermomagnetic recording, the MA facilitated magnetization reversal by applying a magnetic field opposite to the initially magnetized direction of the recording media. In this study, we investigated the effect of MA recording through numerical simulation and experimentation and experimentally demonstrated improvement of the reconstructed images in Bi:RIG.

## Results and Discussion

### Effect of the shape of the non-magnetized region on the stray magnetic field

**Figure 2:**
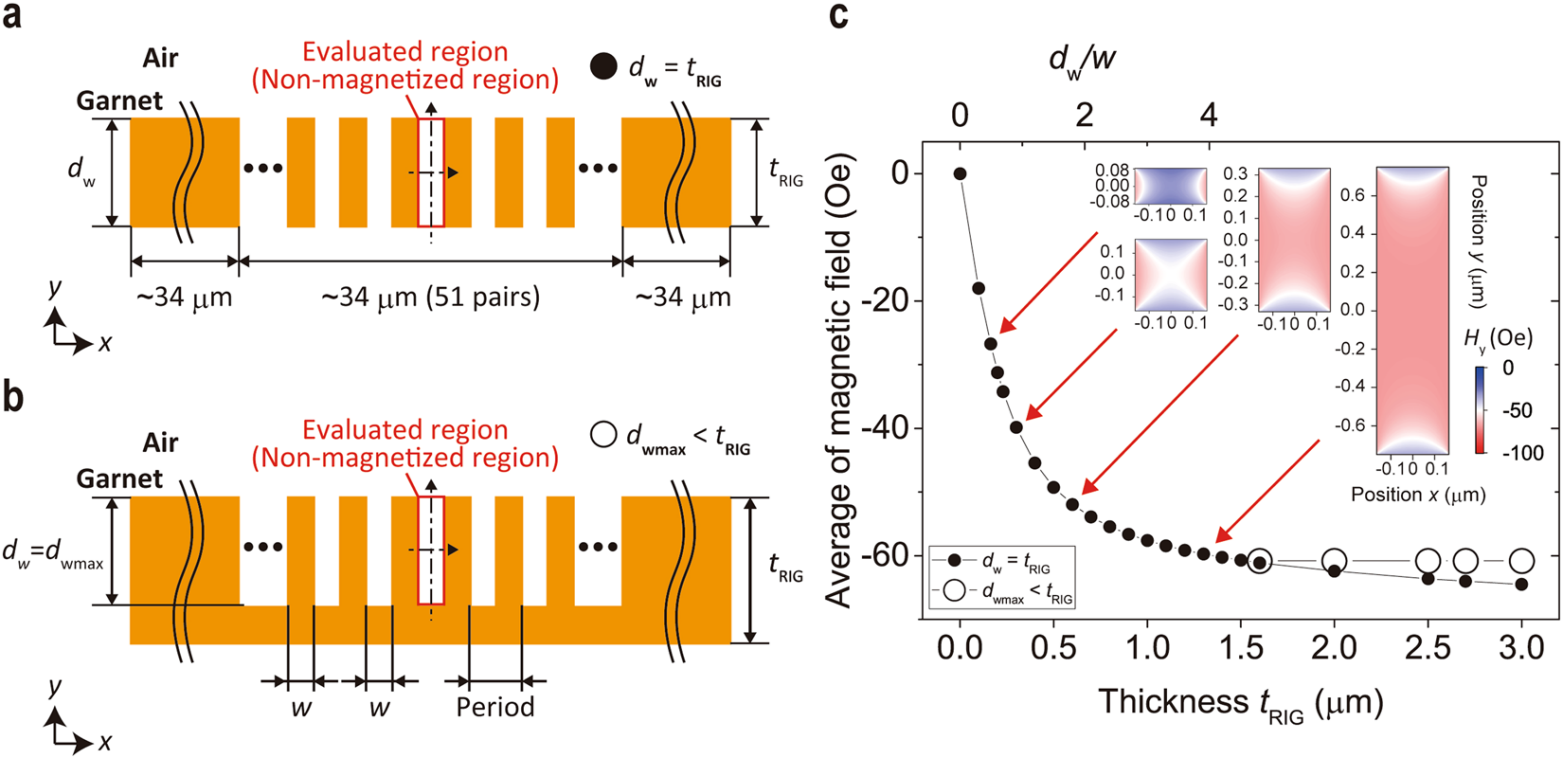
Numerical simulation of a stray magnetic field in a magnetic grating based on a two-beam interference pattern. Calculation models for stray magnetic field (**a**) when *d*_w_ = *t*_RIG_ and (**b**) when *d*_wmax_ < *t*_RIG_. (**c**) The calculated average of the magnetic field at the centre axis of the non-magnetized region. The filled symbols are results for *d*_w_ = *t*_RIG_, and the open symbols are results for *d*_wmax_ < *t*_RIG_. The insets show the distributions of the stray magnetic field in a non-magnetized region with several aspect ratios. The colour represents the intensity of the perpendicular stray magnetic field *H*_y_.

To estimate the intensity of magnetization reversal in a region thermally demagnetized by thermomagnetic recording, we investigated the effect of the shape of the non-magnetized region in the garnet film on the stray magnetic field. The magnetic field distribution was calculated by the finite-element method (COMSOL Multiphysics 4.3a). Figure 2a and b show the calculation models based on a simple two-beam interference pattern with the spatial frequency of 1500 line pair/mm, where the period of the magnetic fringe was $2w=1/1500\times {10}^{3}$ μm with the same width, *w*, of the magnetized and non-magnetized regions. The magnetization of the garnet film was set to be 135 G perpendicular to the surface, and the magnetic field distribution was calculated for several garnet film thicknesses, *t*_RIG_. Because the maximum depth of the magnetic fringe, *d*_wmax_, was reported to be limited to about 1.5 μ=m^13^, a stray magnetic field with a fully magnetized layer below the magnetic fringe was also evaluated while the writing depth was fixed, *d*_w_ = *d*_wmax_ = 1.5 μm in the case of *d*_wmax_ < *t*_RIG_, as shown in Fig. 2b.

Figure 2c shows the calculated average strength of the stray magnetic field along the centreline of the non-magnetized region with respect to the garnet film thickness, where a negative value indicates that the direction of magnetic field is opposite to that of the initial magnetization. The second horizontal axis also shows the aspect ratio, *d*_w_/*w* (where *d*_w_ = *t*_RIG_ for *t*_RIG_ ≤ 1.5 µm), of the non-magnetized region. The insets of Fig. 2c show the distributions of the perpendicular stray magnetic field, *H*_y_, at the non-magnetized region at the centre of magnetic films with several aspect ratios. In the case of *d*_w_/*w* > 2, the absolute value of the stray magnetic field slightly increased as the garnet film thickness increased and almost saturated where *d*_w_/*w* > 4. On the other hand, in the case of *d*_*w*_/*w* < 2, the absolute value of the stray magnetic field decreased rapidly as the thickness of the garnet layer, *t*_RIG_ = *d*_*w*_, decreased. As shown in the insets of Fig. 2c, the strong magnetic field (red coloured) area occupied the non-magnetized region when *d*_w_/*w* > 2, while the weak stray magnetic field (blue coloured) area occupied the most of non-magnetized region when *d*_w_/*w* < 1. The same tendency was obtained for other shapes of the non-magnetized region. That is, the average stray magnetic field was almost saturated when the thickness of the non-magnetized region was more than twice the width of the region, while the average stray magnetic field strength decreased rapidly when the thickness of non-magnetized region was smaller than the width. That is, the absolute strength of stray magnetic field depended on the shape of non-magnetized region, especially when the aspect ratio of non-magnetized region, *d*_w_/*w*, was less than 1. Note that the stray magnetic field at the centre of the non-magnetized region was about half that of the initially magnetized region even when the stray magnetic field was saturated at *d*_w_/*w* > 4. Hence, the effect of MA should be large for garnet films that are thinner than the magnetic fringe period.

### Fundamental properties of Faraday rotation under magnetic field

**Figure 3:**
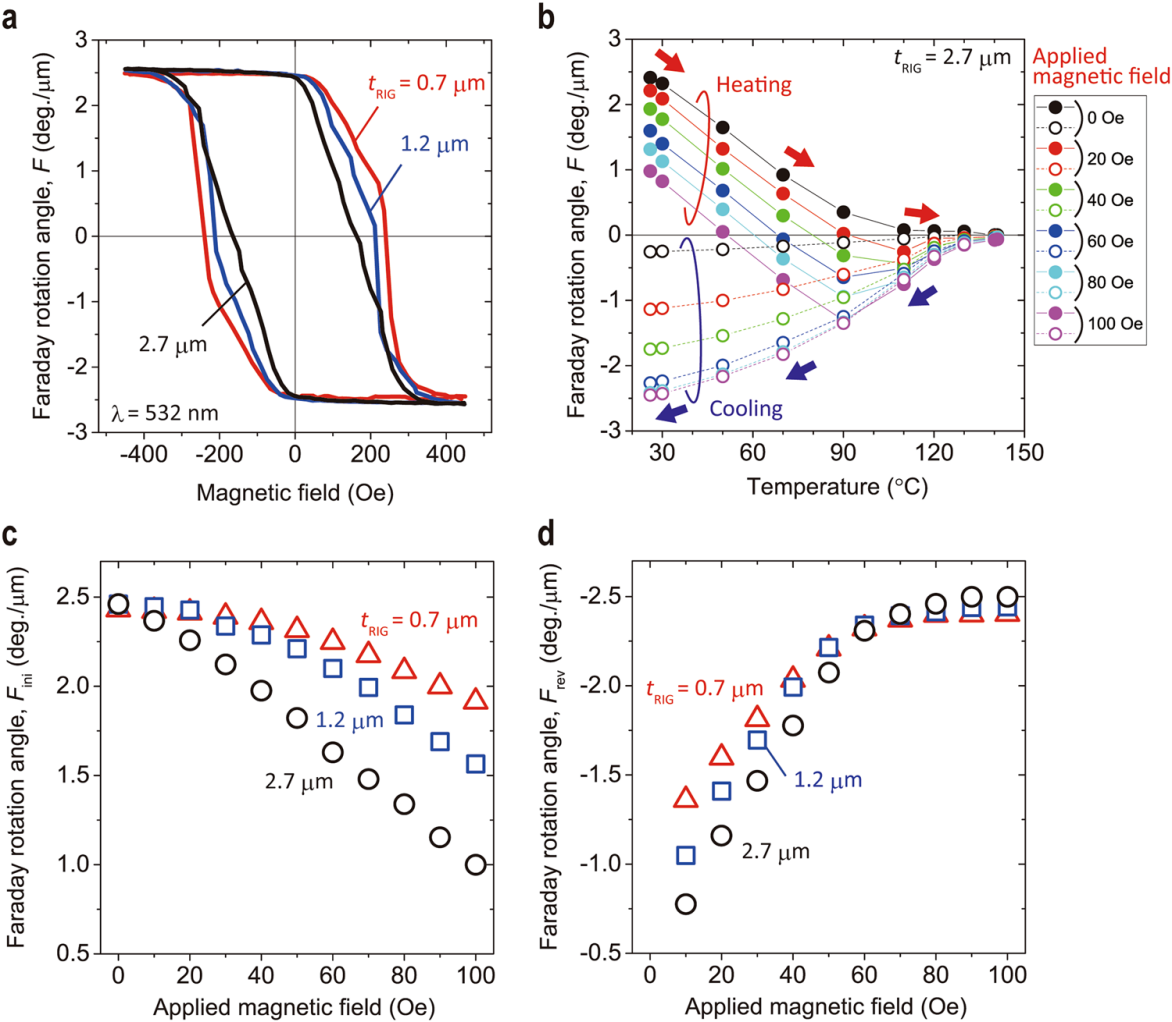
Magnetization properties under applied magnetic field with MO. (**a**) Faraday loop of Bi:RIG films at room temperature with garnet film thicknesses of 0.7 μm, 1.2 μm, and 2.7 μm. (**b**) The temperature dependence of the Faraday rotation angle for the 2.7-µm-thick Bi:RIG film. The garnet film was heated and cooled in the applied magnetic field after magnetization. The magnetic field was applied in the direction opposite to the initially-magnetized direction. (**c**) The Faraday rotation angle of initial magnetization, and (**d**) the Faraday rotation angle of reversed magnetization.

Figure 3a shows the Faraday loops for the fabricated films (see Methods) at a wavelength of 532 nm. The squareness of the Faraday loops differed with the thickness of the Bi:RIG film, and the thin films of *t*_RIG_ = 0.7 µm showed relatively good squareness. The remnant Faraday rotation angle, *F*_r_, of all samples was about 2.45 deg./μm without the magnetic field after saturation.

Figure 3b shows the temperature dependence of the Faraday rotation angle, *F*, of the 2.7-μm-thick Bi:RIG film under various magnetic fields up to 100 Oe that are applied in the direction opposite to the initially magnetized direction. In thermomagnetic recording, the applied magnetic field corresponds to the sum of the stray magnetic field and the assist magnetic field, *H*_assist_, and the heating temperature corresponds to the recording energy. In the heating process, *F* decreased to zero as the temperature increased to 140 °C without the applied magnetic field. On the other hand, in the case of MA recording, the rotation angle, *F*, which decreased by just applying the magnetic field without heating, decreased to negative values as the temperature increased, which depended on the strength of magnetic field, and returned to zero at 140 °C. In the cooling process, the absolute value of *F* monotonically increased and showed a large negative value under higher applied magnetic fields. In MA recording, *F* of initial magnetization corresponded to that before the heating process, and the magnetization of the recorded region should show a negative large value after the heating and cooling processes, depending on the applied magnetic field. Figure 3c shows the magnetic field dependence of *F* before heating, and Fig. 3d shows that after heating and cooling processes for samples with several thicknesses. The Faraday rotation angle before heating, *F*_ini_, decreased as the applied magnetic field increased for all samples, as shown in Fig. 3c, while the reduction of *F* of the thin garnet film was relatively small due to the good squareness of the Faraday loop shown in Fig. 3a. On the other hand, the increase in the applied magnetic field resulted in the increase of the absolute value of *F* after the heating and cooling processes, *F*_rev_, as shown in Fig. 3d. These results indicated that there was a trade-off with MA in the relationship between *F*_ini_ and *F*_rev_. Thus, a suitable value of the *H*_assist_ could be determined to improve diffraction efficiency, and good squareness of the Faraday rotation loop was required for MA recording to suppress the reduction of *F*_ini_.

In addition, after the heating and cooling process, *F* did not reach the same absolute value of *F*_r_ under applied magnetic fields below 60 Oe. According to the calculation results in Fig. 2c the stray magnetic fields were 53.9, 59.1, and 60.8 Oe (*d*_w_ < *t*_RIG_) at *t*_RIG_ = 0.7, 1.2, and 2.7 μm, respectively; these field strengths were obtained ignoring the decrease in the initial magnetization by heat and applied magnetic field. Following these, the strength of reversed magnetization would be about 80 to 90% of the initial value. However, comparisons between the experiment and simulation on the diffraction efficiency^15^ suggested that the stray magnetic field was smaller. For this reason, in the stray magnetic field calculation model, the calculation was performed by giving an ideal interference pattern to the remnant magnetization. In actual thermomagnetic recording, the stray magnetic field may be decreased because of the reduction of the initial magnetization by the increasing temperature due to thermal diffusion in the vicinity of the non-magnetized region. As a result, the actual magnetization reversal may be smaller than expected when the thermal effects were considered.

### Diffraction efficiency with MA

The diffraction efficiency, *η*, of the magnetic hologram is theoretically expressed as

1 $$\eta \propto {\sin }^{2}({F}_{{\rm{fringe}}}{d}_{{\rm{w}}})\approx {({F}_{{\rm{fringe}}}{d}_{{\rm{w}}})}^{2}$$

where *F*_fringe_ is the Faraday rotation angle of magnetic fringes per unit length^10,15^. Here, *F*_fringe_ is defined as the difference between *F*_ini_ and *F*_rev_:

2 $${F}_{{\rm{fringe}}}=\frac{{F}_{{\rm{ini}}}-{F}_{{\rm{rev}}}}{{\rm{2}}}$$

**Figure 4:**
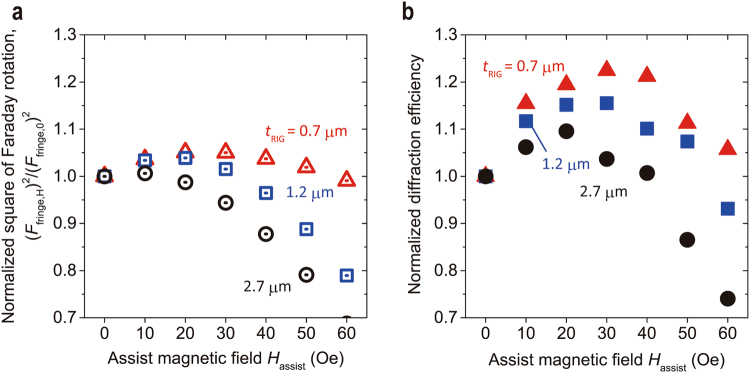
Square of Faraday rotation and diffraction efficiency with MA under various assist magnetic fields normalized with those without MA. (**a**) The assist magnetic field *H*_assist_ dependence of the square of the Faraday rotation evaluated from the specific Faraday rotation angles shown in Fig. 3c and d and the calculated stray magnetic field shown in Fig. 2c normalized with that without *H*_assist_. (**b**) The diffraction efficiency that was normalized without MA experimentally obtained under various *H*_assist_ by two-beam interferometer system.

This value depends on the strength of *H*_assist_ because *F*_ini_ and *F*_rev_ are changed by the applied magnetic field. Based on this relation, the Faraday rotation under magnetic field was calculated from the experimentally obtained properties shown in Fig. 3c and d. Figure 4a shows the square of the Faraday rotation under *H*_assist_, *F*_fringe,*H*_^2^, normalized with that without *H*_assist_, *F*_fringe,0_^2^, which corresponds to the normalized diffraction efficiency, *η*_norm_ $={({F}_{{\rm{fringe}},H})}^{2}/{({F}_{{\rm{fringe}},0})}^{2}$. The normalized *η*_norm_ was increased by applying a magnetic field and showed the maximum value at *H*_assist_ = 10, 20, and 30 Oe at garnet film thicknesses of 0.7 μm, 1.2 μm, and 2.7 μm, respectively. This increase was the result of the increase of *F*_rev_, caused by *H*_assist_ as shown in Fig. 3d. After *η*_norm_ reached its maximum value, *η*_norm_ dwecreased with the increasing *H*_assist_ as a result of the reduced *F*_ini_ through the decrease of the initial magnetization. Therefore, an increase of *F*_rev_ that is greater than decrease of *F*_ini_ was required to improve *η*. As a result, the improvement of *η* was expected to be larger in thinner garnet films because thinner films have low stray magnetic field strength and had good Faraday loop squareness.

**Figure 5:**
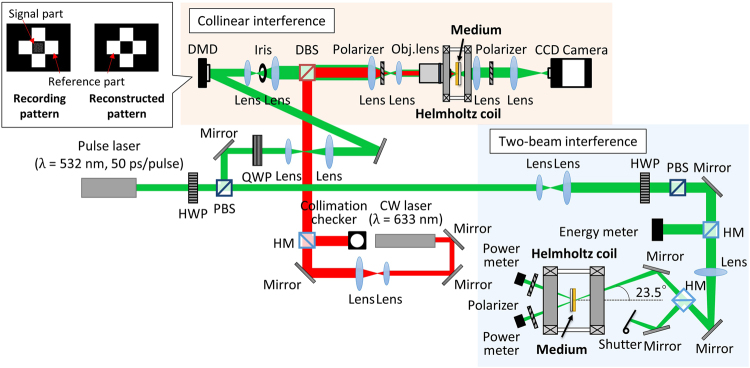
Schematic illustration of the optical setup for collinear and two-beam holography. A pulsed laser (λ = 532 nm and a pulse width of 50 ps) is used for recording and reconstructing. The CW laser (λ = 633 nm) is used to position the recording media at the focal length of the objective lens. In the collinear interference system, the recording pattern modulated with a DMD is divided into two parts: the signal part and the reference parts, and only the reference parts are used for reconstruction. DMD: Digital mirror device; HWP: Half-wave plate; QWP: Quarter-wave plate; PBS: Polarizing beam splitter; DBS: Dichroic beam splitter; HM: Half mirror.

The two-beam interferometer system schematically shown in Fig. 5 was used to evaluate *η* in Bi:RIG (Method). The experimentally evaluated *η* without *H*_assist_, *η*_0_, the maximum *η* with *H*_assist_, *η*_H_, and the improved ratio, *η*_H_/*η*_0_, by MA for each Bi:RIG are summarized in Table 1. The experimentally evaluated value of *η* normalized with *η*_0_ is shown in Fig. 4b against *H*_assist_. The change in normalized *F* showed the same trend as that shown in Fig. 4a. The maximum improvement in the *η* was achieved in *t*_RIG_ = 0.7 µm, as expected from the calculation. This improvement of *η* could be explained by improvement of *F* of magnetic fringes, and the effect of MA depends on the squareness of the Faraday loop and the strength of the stray magnetic field. In this recording case, the improvement of *η* by MA was larger than expected, as shown in Fig. 4a, probably because the actual magnetization reversal was smaller. The experimentally evaluated *η* and calculated *η*_norm_ did not quantitatively agree because the evaluated strength of the stray magnetic field did not take into account the thermal effects.

**Table 1:** The experimentally obtained diffraction efficiency by MA.

Thickness *t*_RIG_ (µm)	Diffraction efficiency without MA, *η*_0_ (%)	Maximum diffraction efficiency with MA, *η*_H_ (%)	Maximum improvement rate by MA, *η*_H_/*η*_0_ (%)
0.7	1.7 × 10^−3^	2.1 × 10^−3^	22.5
1.2	5.8 × 10^−3^	6.7 × 10^−3^	15.5
2.7	25.0 × 10^−3^	27.4 × 10^−3^	9.6

### Reconstructed image in collinear interferometer with MA

The collinear interferometer system schematically shown in Fig. 5 was used to record and reconstruct magnetic holograms in Bi:RIG (Method). We prepared the recording patterns of page data and a reference beam and displayed these on a digital mirror device (DMD). The reconstructed images were obtained by radiating only the reference beam onto the recording medium, and the error ratio (ER) was evaluated by using the following equation:

3 $$ER=\frac{{N}_{{\rm{error}}}}{{N}_{{\rm{page}}}}\times 100\,( \% )$$

where *N*_page_ is the total number of pixels in signal region, and *N*_error_ is the number of error pixels, respectively.

**Figure 6:**
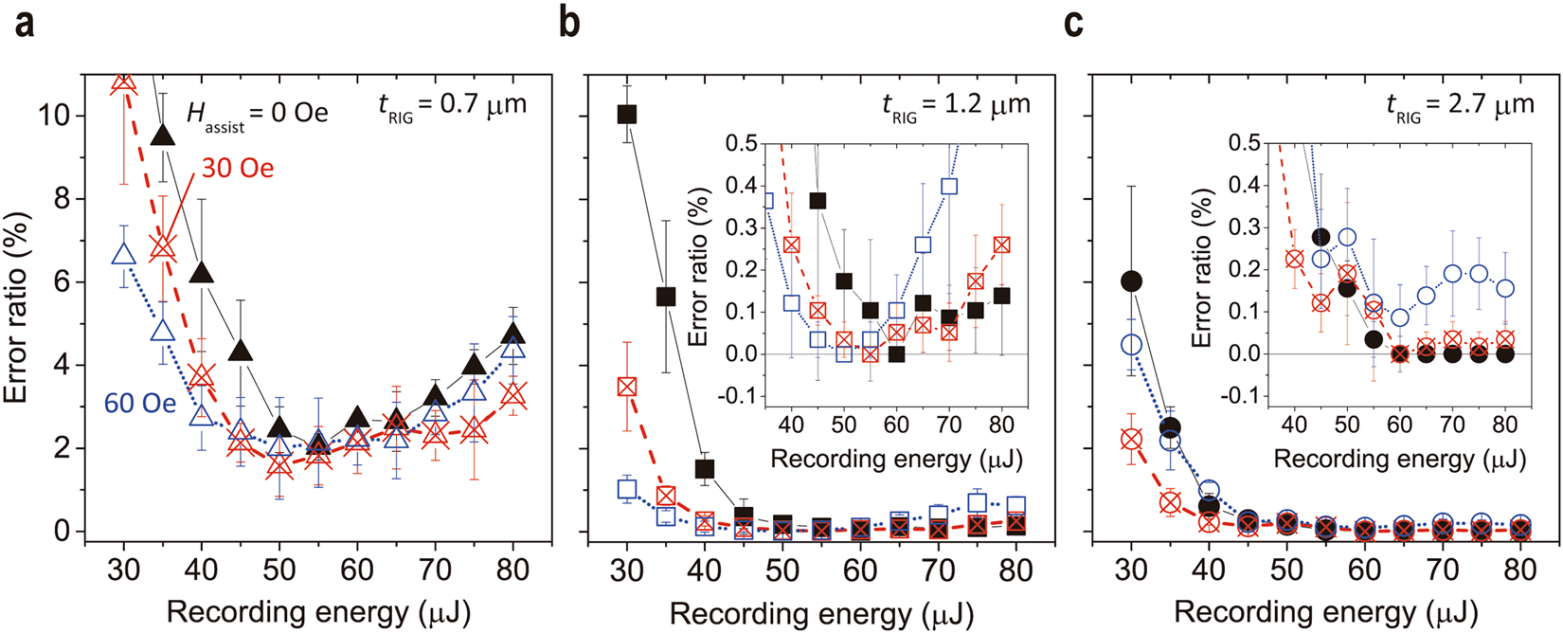
Recording energy dependence of error ratio with MA under various assist magnetic fields. The inset shows the enlarged view of the part where the error rate is small. The ER of Bi:RIG films with (**a**) *t*_RIG_ = 0.7 μm, (**b**) 1.2 μm, and (**c**) 2.7 μm experimentally obtained by the collinear interferometer system.

**Figure 7:**
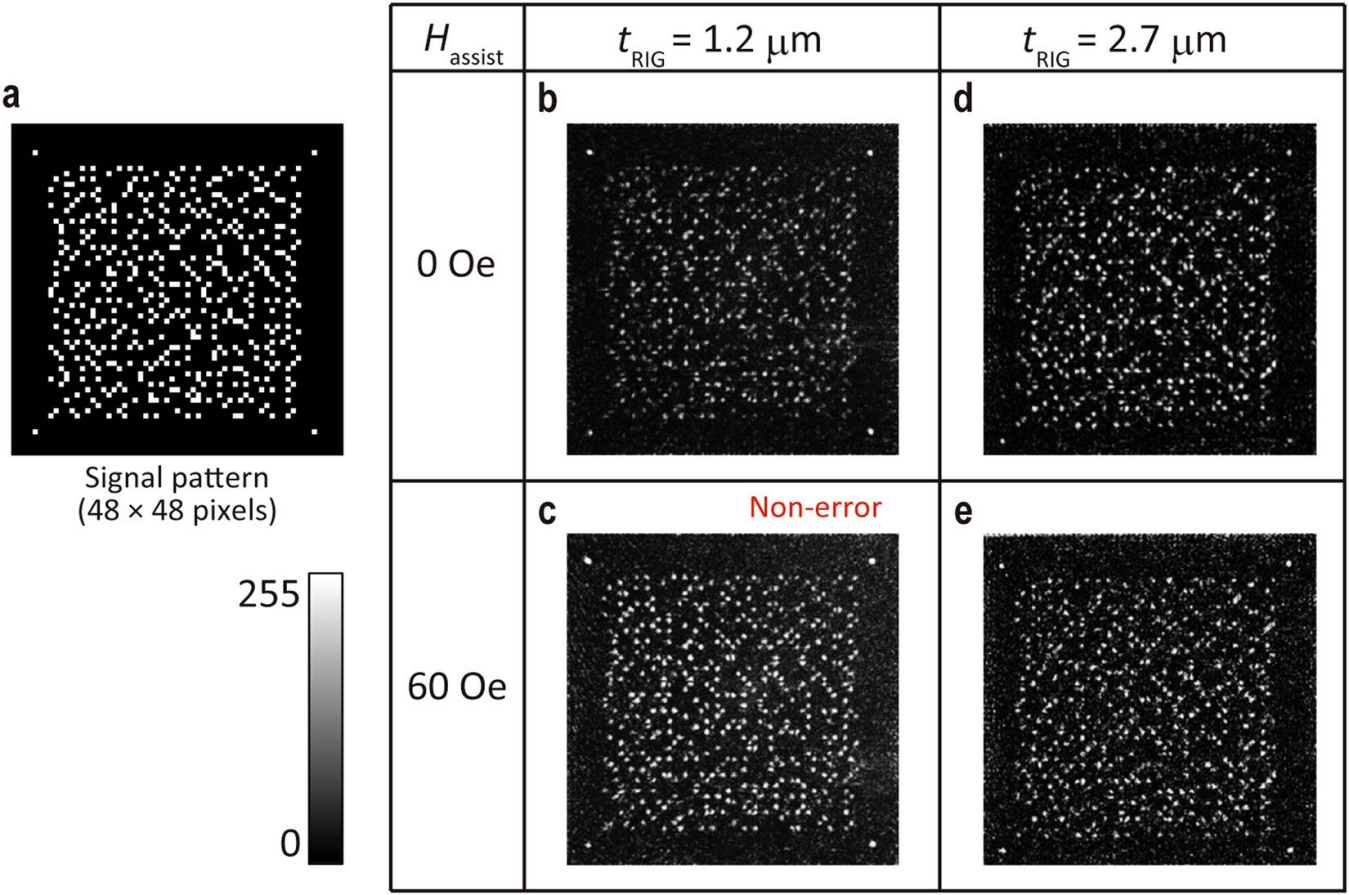
Reconstructed images of signal pattern. (**a**) Design of the signal pattern. The reconstructed images from garnet films with *t*_RIG_ = 1.2 μm corresponding to (**b**) non-MA and (**c**) MA, and with *t*_RIG_ = 2.7 µm corresponding to (**d**) non-MA and (**e**) MA (Recording energy = 40 µJ). The greyscale represents the brightness of images.

Figure 6 shows the relation of ER with respect to the recording energy under several film thickness and magnetic fields. The ER was decreased with increasing recording energy and became 0 at recording energies over 60 µJ without MA at *t*_RIG_ = 1.2 μm and 2.7 μm, as shown in Fig. 6. The ER in *t*_RIG_ = 0.7 µm was improved by MA; however, the ER did not reach non-error status (*ER* = 0) due to low *η*. On the other hand, the ER of reconstructed images in *t*_RIG_ = 1.2 µm and 2.7 µm films recorded with MA was smaller than those recorded without MA, even in lower energy regions (<50 µJ). Figure 7 shows the reconstructed images with and without MA at the recording energy of 40 µJ. Obviously, the reconstructed image in *t*_RIG_ = 1.2 µm with MA (*H*_assist_ = 60 Oe that was condition when the lowest ER in this recording energy) was brighter than that without MA. In other words, the magnetization of the heated region was easily reversed under the *H*_assist_ at the low recording energy condition because the larger applied magnetic field decreased the heating energy required for thermal demagnetization, as shown Fig. 3b. In addition, magnetization reversal at low temperature was favourable for maintaining the high initial magnetization, which led to keep high *η*. As a result, the intensity of the reconstruction images was enhanced through the improvement of *η*, and the non-error recording conditions were broadened to the low energy region. On the other hand, in the case of *t*_RIG_ = 2.7 µm, the MA improvement was small due to the bright image without MA, and, on the contrary, the ER increased under *H*_assist_ = 60 Oe despite the brighter reconstructed image because the ON signals (white part) disappeared or the background noise was also increased with MA. This small improvement would be explained by the large magnetization reversal without MA suggested through numerical simulation and also by the decrease in the initial magnetization by MA due to the small squareness of *F*, which may lead to misshapen magnetic fringes. There were suitable values of the *H*_assist_ corresponding to the recording media for producing clear reconstructed images as well as for achieving high *η* with MA.

## Conclusion

To improve the reconstructed images of the magnetic hologram, we investigated the effect of the thermomagnetic recording using MA. First, the stray magnetic field distribution was calculated for the non-magnetized region, and we found that the intensity of the stray magnetic field depended on garnet film thickness or substantially on the aspect ratio of the non-magnetized region. Second, the magnetic field dependence of Faraday rotation angle of initial and reversed magnetization was evaluated experimentally. By applying a magnetic field opposite to the initial magnetized direction, the initial magnetization was decreased, and the reversed magnetization was increased. Based on these results, *η* was evaluated with MA, and the peak value depended on the balance of the Faraday rotation between the reduction of the initial magnetization and the increase of the reversed magnetization in the magnetic fringes. In this experiment, the *η* of *t*_RIG_ = 0.7 µm was improved more than that of *t*_RIG_ = 2.7 µm due to the small stray magnetic field and the good Faraday loop squareness in the thinner films, while the *η* of *t*_RIG_ = 2.7 µm was still larger than that of *t*_RIG_ = 0.7 µm. Finally, the reconstructed images produced with MA recording were especially improved in low energy recording conditions because MA recording decreased the recording energy necessary for magnetization reversal. The lowest recording energy required for non-error reconstruction was decreased from 60µJ to 45 µJ by MA recording in *t*_RIG_ = 1.2 µm. As a result, the brightness of the reconstructed image was improved due to the improved *η*, and non-error reconstruction was achievable under broad, low-energy recording conditions with a suitable MA value. We therefore note that MA recording can produce clearer reconstructed images with the collinear interference system and improve the reconstruction quality.

## Methods

### Sample preparation

The Bi:RIG films were deposited on nonmagnetic substituted gadolinium gallium garnet (SGGG) substrates by radio-frequency magnetron sputtering with an oxide target composition of Bi:Dy:Y:Fe:Al = 1.5:1.0:1.0:3.8:1.2. The deposited films were crystallized by rapid thermal annealing at 750 °C for 15 min in air because the as-deposited films were not crystallized. To observe the effect of MA on the garnet film thickness, we prepared three samples with garnet film thicknesses of 0.7 μm, 1.2 μm, and 2.7 μm. The thicker film is desirable for its high *η* and volumetric recording, whereas a thinner film is suitable for multilayer structures such as the magnetophotonic microcavity (MPM) structure^10,16,17^. The Faraday loop of these samples was evaluated using a rotating analyser method. In addition, the magnetic field dependence of the Faraday rotation angle was also characterized at several temperatures up to 140 °C to observe the fundamental properties under magnetic field strengths from 0 to 100 Oe.

### Optical setup

We evaluated *η* experimentally using the two-beam interference system shown in Fig. 5. A pulsed laser with a wavelength λ of 532 nm and a pulse width of 50 ps was used for recording and reconstructing. The beams were tilted 23.5° from the normal direction of the surface of the recording media so that holograms with a spatial frequency of 1500 line pair/mm were formed. The recording media were placed in the centre of a Helmholtz type electromagnet for MA recording. We recorded interference patterns with and without a magnetic field that was varied from 0 to 60 Oe in the direction opposite to the initial magnetization direction and evaluated *η*.

In this work, the diffraction efficiency, *η*, was evaluated using the following equation^13^:

4 $$\eta =\frac{{I}_{1}}{{I}_{{\rm{0}}}+{I}_{{\rm{1}}}}\times 100\,( \% )$$

where *I*_0_ is the zero-order transparent light intensity, and *I*_1_ is the first-order diffracted light intensity, respectively. The value of *η* was evaluated with several recording energies for each *H*_assist_.

The collinear interference system schematically shown in Fig. 5 was used to record and reconstruct magnetic holograms in Bi:RIG. A pulsed laser with a wavelength of 532 nm and a pulse width of 50 ps was divided into a signal part and reference parts using a DMD. The recording media were located near the focal point. Interference patterns were recorded by the MA recording method under magnetic fields from 0 Oe and 60 Oe. We used 48 × 48 pixels with 3:16 encoding methods^18^ for the signal.
